# Supplementary material for: Robust high-dimensional memory-augmented neural networks
Source: Nat Commun. 2021 Apr 29;12:2468. doi: 10.1038/s41467-021-22364-0 (PMC8084980; doi:10.1038/s41467-021-22364-0)
Supplement: Supplementary file 1 — Supplementary Information [file 41467_2021_22364_MOESM1_ESM.pdf]

Supplementary Information

Robust High-dimensional Memory-augmented Neural Networks

Geethan Karunaratne,[1,2,a] Manuel Schmuck,[1,2,a] Manuel Le Gallo,[1] Giovanni Cherubini,[1] Luca Benini,[2] Abu Sebastian,[1,b] and Abbas Rahimi[1,c]

[1] *IBM Research – Zurich, Säumerstrasse 4, 8803 Rüschlikon, Switzerland.*

[2] *Department of Information Technology and Electrical Engineering, ETH Zürich, Gloriastrasse 35, 8092 Zürich, Switzerland.*

[a] These two authors contributed equally

[b] Electronic mail: ase@zurich.ibm.com

[c] Electronic mail: abr@zurich.ibm.com

SUPPLEMENTARY FIGURES

Supplementary Figure 1: Architecture to implement bipolar key memory using PCM crossbar arrays

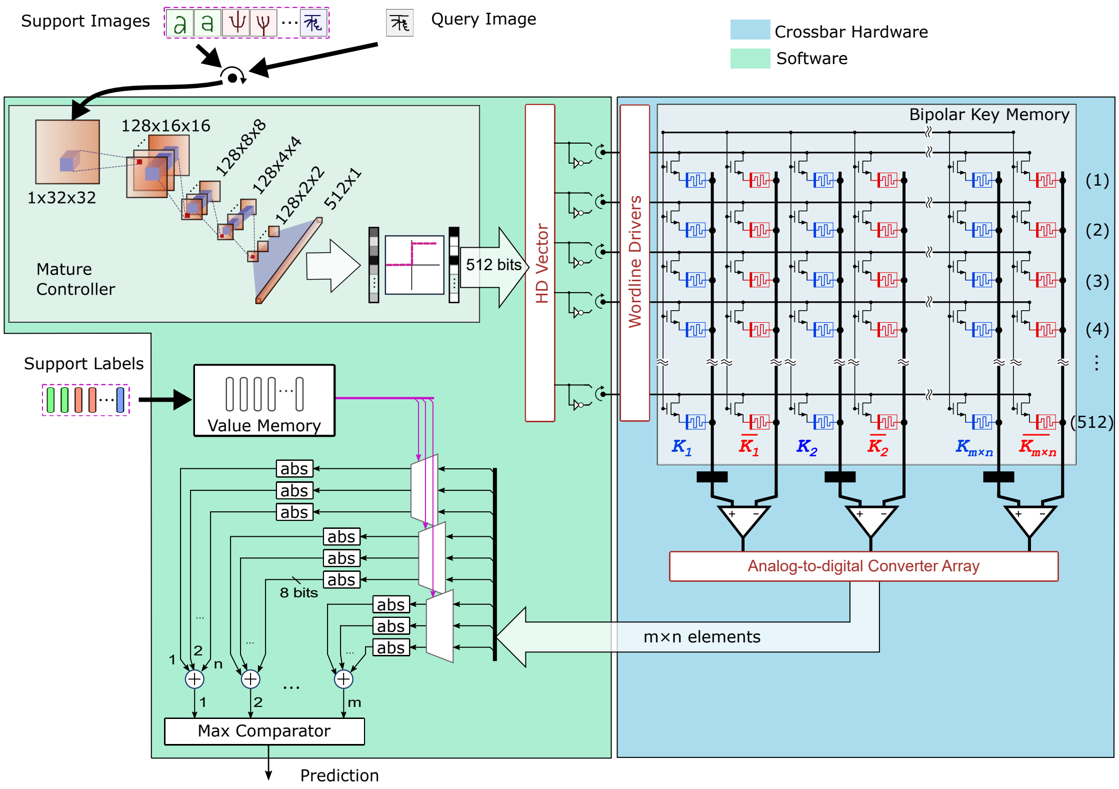

Supplementary Figure 1: The MANN architecture with the bipolar key memory using analog in-memory computations. This architecture is different from the one presented in Fig. 3 in the main manuscript for the binary representations in the following ways: First, the activation function used at the output of the embedding function in the controller is changed to a sign function, generating bipolar query and support vectors. Second, the crossbar utilizes twice the number of columns compared to the binary architecture to store the complementary versions of the support vectors on the crossbar. This effectively doubles the number of memristive devices. Third, a regular absolute (abs) function approximates the softabs sharpening function during inference. Fourth, there are some changes to the peripheral circuits in the way the original support/query vector are fed from the controller: the complementary version of it is fed to the wordline drivers in a time multiplexed manner. Furthermore, the resulting current on the bitline from the original support vectors is saved in an array of capacitors and subtracted from the current measured on the corresponding complementary bitline before sending the net current to the analog-to-digital converter array. The blue columns represent the original support vectors, whereas the red columns indicate the complementary versions.

Supplementary Figure 2: Robustness of bipolar versus binary architectures

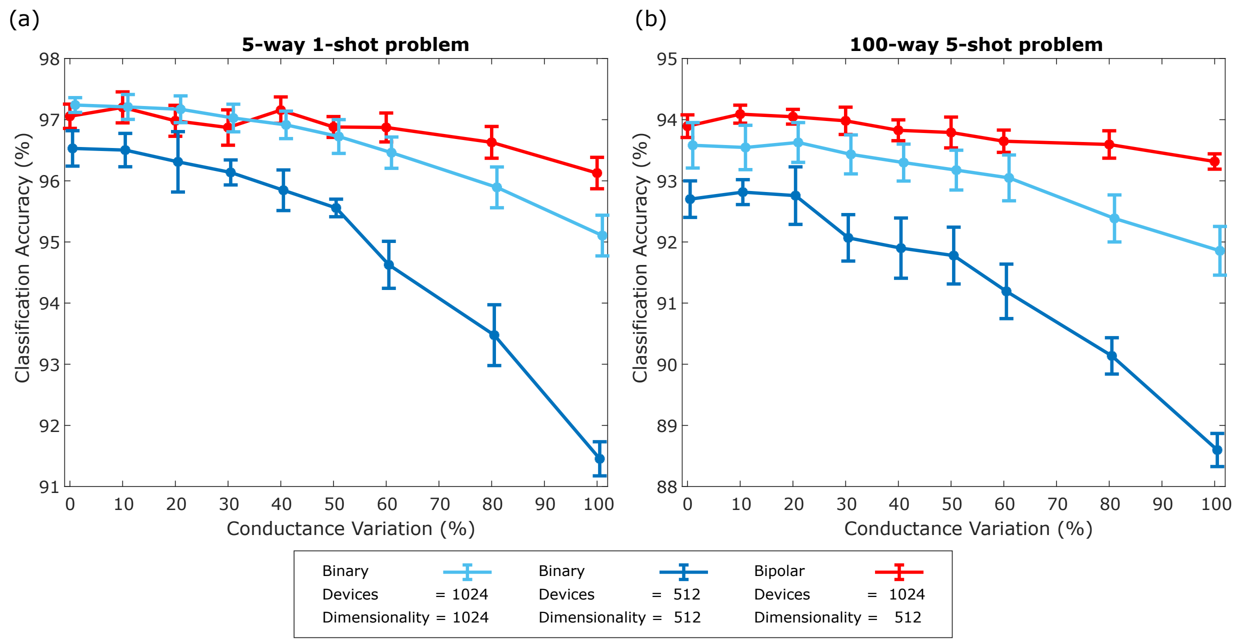

Supplementary Figure 2: Classification accuracy when using the PCM model as a function of device conductance variations for the 5-way 1-shot **(a)** and the 100-way 5-shot problems **(b)**. The bipolar architecture (with dimensionality $d = 512$ and hence 1024 devices) is compared against the binary architecture with i) the same dimensionality ($d = 512$), and ii) the same number of devices ($d = 1024$). At zero or low conductance variations, the binary architecture with 1024-dimension outperforms the same architecture with 512-dimension. This indicates a loss of information when the representation dimensionality is lowered from 1024 to 512. This loss is orthogonal to the loss incurred by non-idealities because conductance variation is on the lower side. The bipolar architecture on the other hand achieves a relatively higher accuracy at the lower dimensionality of 512 and retains it even when extreme conductance variations are presented, compared to the both binary architectures with the same dimension, or the same number of devices. This implies that the bipolar architecture is more robust against non-idealities of in-memory computing for the same power and area constraint. The accuracy distributions were obtained from 8 test runs each containing 1000 episodes. The error bars represent one standard deviation of sample distribution on either directions.

Supplementary Figure 3: Robustness of similarity measurement for different vector dimensionalities

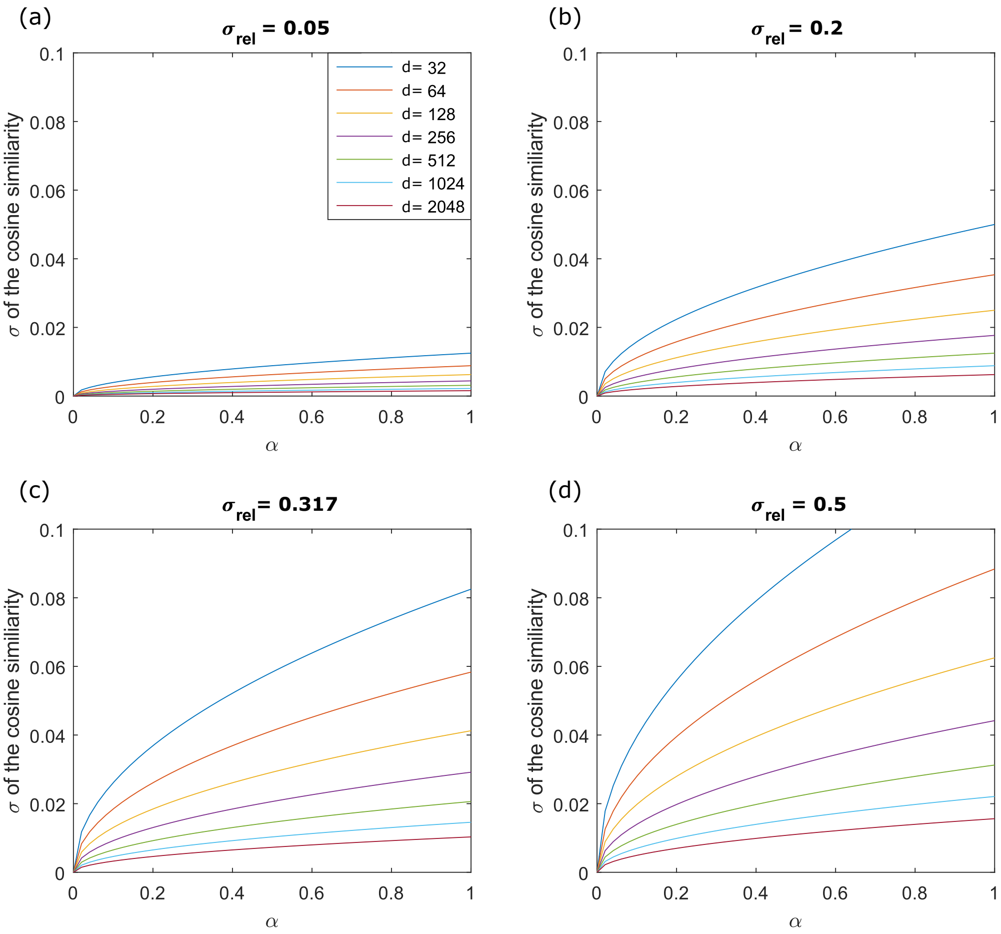

Supplementary Figure 3: Theoretic deviations in the computed cosine similarity for different vector dimensionalities d, and different SET state variabilities σ_{rel}. The case $\sigma_{\text{rel}} = 0.317$ represents the variability observed on our PCM crossbar array as shown in Supplementary Note 6. In this case, the uncorrelated binary vectors (i.e., $\alpha = 0.5$) exhibit merely ≈ 0.015 standard deviation in the measured cosine distance when using 512-bit vectors.

Supplementary Figure 4: PCM Measurements and Model Fitting

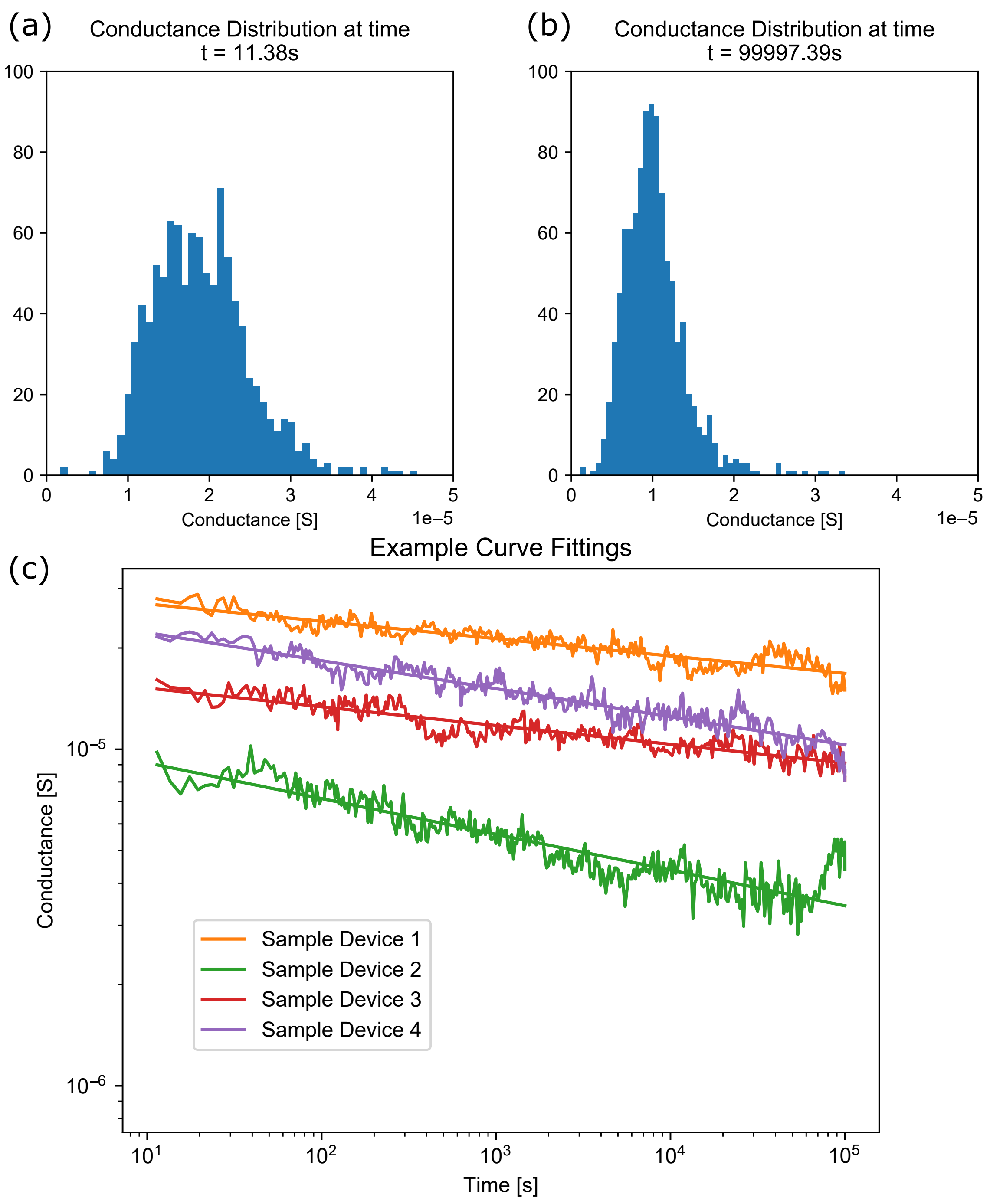

Supplementary Figure 4: Conductance distribution of 10,000 devices in the SET state at the beginning ($\sigma_{\text{rel}} = 31.7\%$) **(a)** and at timescales three orders of magnitude later ($\sigma_{\text{rel}} = 36.6\%$) **(b)** of the experiment. SET state measurements from 4 example devices, with their fitted curves **(c)**.

SUPPLEMENTARY TABLES

Supplementary Table 1: PCM model parameters

Supplementary Table 1: Derived values of the model parameters.

Symbol	Description	Type	Value
G_0	mean conductance at time $t = 1s$	-	22.8×10^{-6} S
ν	mean drift exponent	-	0.0598
$\tilde{G}_{\mathrm{p}}$	programming variability	multiplicative	31.7 %
$\tilde{G}_{\mathrm{r}}$	read-out noise	additive	0.496×10^{-6} S
$\tilde{\nu}$	drift variability	multiplicative	9.07 %

SUPPLEMENTARY NOTES

Supplementary Note 1: The CNN Controller in Conformity with Dense High-dimensional Representations

We evaluate our training methodology to verify whether the trained CNN controller obeys the "laws" of high-dimensional computing. Specifically, the pseudo-randomness of the dense representations is crucial for our findings to be valid. In the theory of high-dimensional computing[2], the ratio between the number of -1s and 1s in the bipolar vectors (respectively, 0s and 1s in the binary vectors)—termed occupancy ratio—closely approximates at $\frac{1}{2}$. This holds when the components of a vector are drawn randomly from $\{-1, 1\}$ (respectively $\{0, 1\}$) with equal probability.

In order to verify, whether our controller conforms with this property, we calculate the occupancy ratio for the embeddings of multiple Omniglot samples for different dimensionalities. Supplementary Figure 5 shows the mean and standard deviation of the occupancy ratio at different dimensionalities together with their target value according to the high-dimensional computing theory. While the mean should be constant at $\mu = 0.5$, the standard deviation is dependent on the dimensionality d, in fact $\sigma = 1/(2\sqrt{d})$. As shown, by increasing the dimensionality ($d \geq 512$), the controller generates vectors that closely follow the equiprobable property of vectors in the high-dimensional computing theory. We particularly select $d = 512$, as it also provides sufficient resiliency against the variations in the PCM chip as shown in Supplementary Figure 3, and leads to comparable accuracy with the real-valued vector representations as shown in Supplementary Figure 6.

To show that the distribution is as desired and approximately Gaussian, the distribution of the occupancy ratios for embeddings at $d = 512$ is shown in Supplementary Figure 7. We observe that the vector representations generated by the controller is in conformity with the high-dimensional computing theory, but the means of the occupancy ratios are slightly off. There is 2.08% standard deviation in the occupancy ratio of the controller at $d = 512$. This deviation causes 1.02% accuracy drop (93.97% vs. 92.95%) for the 100-way 5-shot problem

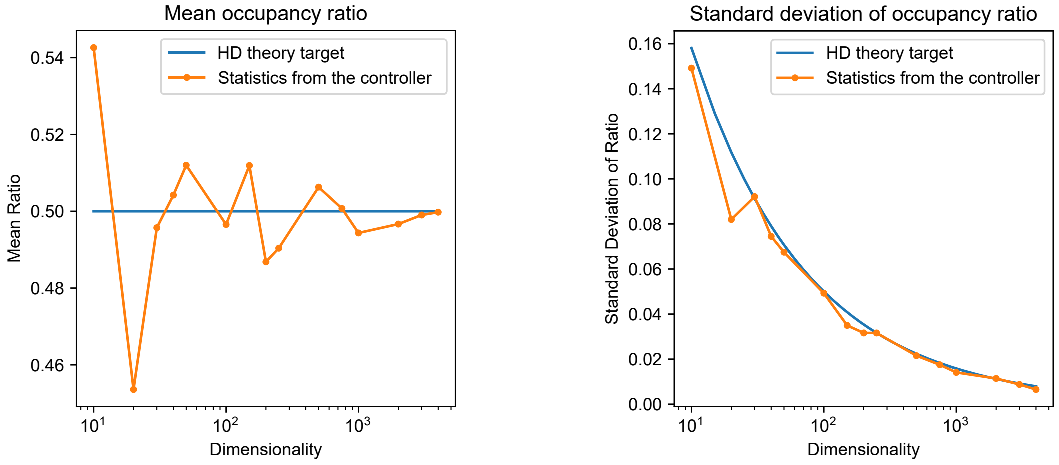

Supplementary Figure 5: Occupancy ratio of the embeddings generated from the CNN controller at different dimensionalities versus the high-dimensional (HD) theory target: Mean (left) and standard deviation (right) of the occupancy ratio.

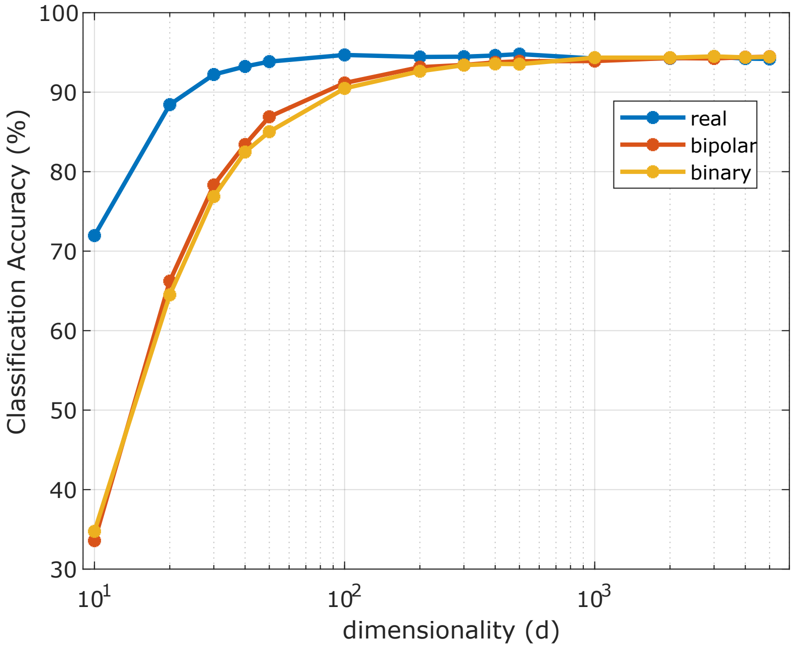

Supplementary Figure 6: Classification accuracy of 100-way 5-shot problem as a function of dimensionality for software models three different representations:real, bipolar, and binary. The results averaged from 3 independent runs (3000 episodes).

in the case of binary representation when the similarity metric is approximated by the dot product as shown in Supplementary Figure 13. We therefore seek an algorithmic solution to reduce the deviation of the occupancy ratio from the desired value in the following.

Introducing a regularizer to optimize the occupancy ratio

In order to penalize a controller for generating output vectors with an occupancy ratio that deviates from the desired value, a regularizing term is introduced into the loss function:

$$\mathcal{L}_{oc} = -\frac{1}{mn}\sum_{i=1}^{mn}(\frac{1}{d}\sum_{j=1}^{d}(\frac{1}{2}\,tanh(a\mathbf{K}_i(j)) + \frac{1}{2}) - 0.5)^2 \tag{1}$$

where the function $\frac{1}{2}\,tanh(ax)+\frac{1}{2}$, also known as softstep, is a differentiable smoothed version of the step function. This loss is minimized when the occupancy ratio is 0.5, in other words, the number of positive vector components ($\mathbf{K}_i(j) > 0$) is equal to the number of negative vector components ($\mathbf{K}_i(j) < 0$), for the ith support vector, hence leading to the desired occupancy ratio. However, there is another condition that can alternatively minimize the loss function by driving the support vector components towards the origin ($\mathbf{K}_i(j) = 0$). It was observed that the controller may reach this undesired condition, which sets the value of support vector components near 0, because the softstep function reaches the same target occupancy ratio of 0.5 when all support vector components approach 0.

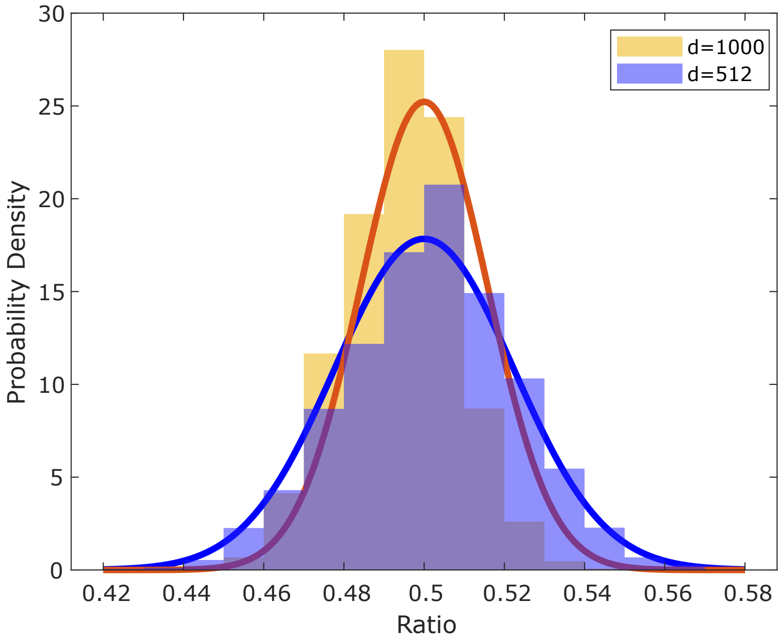

Supplementary Figure 7: Distribution of the occupancy ratios of embeddings for two different dimensionalities, normalized so that they form a probability density function (PDF). Their target PDFs are shown as solid lines.

To avoid this undesired condition, an auxiliary term is added to the loss function:

$$\mathcal{L}_{aux} = -\frac{1}{mnd}\sum_{i=1}^{mn}\sum_{j=1}^{d}(\frac{1}{2}(tanh(a(\mathbf{K}_i(j)+\delta))+1)-\frac{1}{2}(tanh(a(\mathbf{K}_i(j)-\delta))+1)) \quad (2)$$

where parameters a and δ in Supplementary Equation 1 and Supplementary Equation 2 are chosen as 100 and 0.0001, respectively, based on the distribution of real-valued support vectors elements $K_i(j)$. The loss term $\mathcal{L}_{oc}$ is assigned a weight value of 10, and the loss term $\mathcal{L}_{aux}$ is assigned a weight value of 0.1 to keep these losses in a comparable range as with the original steady state log loss given in Supplementary Equation 3.

After introducing the above regularizing terms, the output of the controller conforms more closely with the behavior demanded by the high-dimensional computing theory. For example, the standard deviation of the occupancy ratio from the target 0.5 dropped from 2.08% to 0.91% for $d = 512$. That is effectively equivalent to the standard deviation of pseudo randomly generated vectors for $d \approx 3000$. As a result, the accuracy is consistently increased across all three problems, up to 0.74%, by using the regularizer as shown in Supplementary Table 2. We have shown that the binary architecture using the regularizer and the dot product can almost reach to the accuracy obtained from the cosine similarity without using the regularizer (maximum 0.28% lower accuracy); see Supplementary Table 2. Supplementary Figure 8 also compares the resulting occupancy ratio with and without the regularizer.

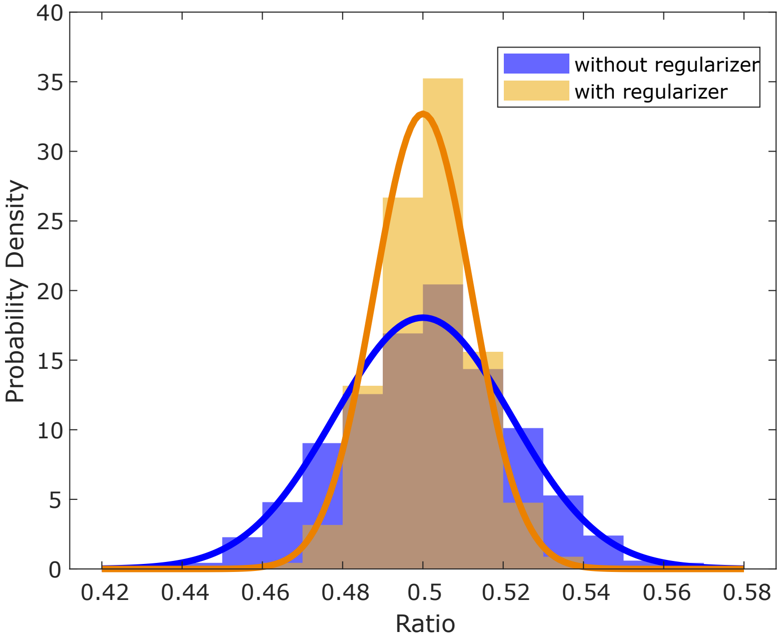

Supplementary Figure 8: Distribution of the occupancy ratios of embeddings for $d = 512$ with and without regularizer, normalized so that they form a probability density function (PDF). Expected respective PDFs with and without regularizer is shown as a line.

Supplementary Table 2: Comparison of average classification accuracy (%) from 10000 episodes (10 runs) with and without using the regularizer

**Problem**	**without regularizer**		**with regularizer**
	**cosine similarity**	**dot product similarity**	**dot product similarity**
5-way 1-shot	97.44	96.92	97.26
20-way 5-shot	97.79	97.38	97.92
100-way 5-shot	93.97	92.95	93.69

Supplementary Note 2: Training and Inference for Key-Value Memory Network

In the following, we describe two main phases in detail, the learning phase and the inference phase, for our proposed MANN architecture. For a summary, refer to Supplementary Table 3 that shows the steps of each phase.

1. Learning Phase

The learning phase starts by randomly initializing the trainable parameters $\boldsymbol{\theta}$ of the embedding function $f_{\boldsymbol{\theta}}$ of the controller. Randomness is important for the feature vectors to adopt certain important properties of high-dimensional computing. For example, the number of positive and negative components of the feature vectors should be approximately equal. The learning phase includes the following steps.

a. Support set loading step The step after initializing the parameters is the very first training step. For that, we (randomly) draw a support set[1] from the training dataset, which is then mapped to the feature space via the controller and stored in the key memory. More specifically, this step generates support vectors from the forward pass through the

Supplementary Table 3: Summary of the learning and inference phases.

	State Before		State After	
	Controller	Key-value Memory	Controller	Key-value Memory
**Learning Phase:**				
Support set loading step[a]	Immature	Empty/Obsolete	Unchanged	Rewritten
Query evaluation step[b]	Immature	Written	Unchanged	Unchanged
Backpropagation step[c]	Immature	Written	More Mature	Unchanged
Episodic training by repeating[d]	Slightly Mature	Written	Mature	Repeatedly Rewritten
**Inference Phase:**				
Support set loading step[e]	Mature	Empty/Obsolete	Unchanged	Rewritten
Query evaluation step[f]	Mature	Written	Unchanged	Unchanged

[a] Support set from training dataset to fill the key-value memory
[b] Query batch from training dataset to evaluate predictions
[c] Loss computed based on classification errors in query phase and backpropagation
[d] The three above steps are repeated by randomly redrawing support sets and query batches from training dataset
[e] Support set from test dataset to fill the key-value memory
[f] Query batch from test dataset

[1] In correspondence with the common machine learning terms, the word "set" and "batch" are sometimes used interchangeably. In this work, "set" will refer to a more general, mathematical construct, whereas "batch" will denote a bundle of data that are processed together. Often, a whole set is processed in one batch.

controller, and writes them in the key memory. Optionally, the dataset training samples can be augmented by shifting and rotating the symbols to improve the learned representations, as we described in the Methods. Each class in the support set gets assigned a unique one-hot label and for each support vector in the key memory, the corresponding one-hot support label is stored in the value memory (see Supplementary Figure 9a). After this step, both key and value memories have been written and will remain fixed until the next training episode is presented (see Supplementary Figure 9b). Henceforth, one can query arbitrarily often without altering the architecture's state at all. It should be emphasized that, as we have just initialized the parameters, predictions will be random, because the controller is still immature.

b. Query evaluation step During one episode of the learning phase, a whole batch of query samples is processed together and later produces a single loss value. There is a maximum size for the query batch, which is dependent on the number of available samples per class in the training dataset. As the query samples stem from the same classes as the samples in the support set, problems with a higher number of shots leave fewer samples for the query batch. Then, the query batch is mapped to the feature space in the same way as the support set. This yields a batch of probability distributions over the potential labels as shown in Supplementary Figure 9c.

c. Backpropagation step This step has to be supervised, i.e., the labels of the query batch need to be available. From the ground truth one-hot labels $\mathbf{Y}$ and the output of the previous step $\mathbf{P}$, the logarithmic loss $\boldsymbol{\lambda}_i$ is computed for every query $i \in \{1, ..., b\}$. It is important to employ the logarithmic loss instead of the cross-entropy loss (i.e., including the additional second term in Supplementary Equation (3)) so that vectors from different classes are pushed further apart. The average loss (Supplementary Equation (4)) represents the objective function that has to be minimized using an appropriate optimization algorithm[2]. Notice that only the controller is affected in this step by backpropagating errors through all modules using the chain rule, while the memories remain fixed as shown in Supplementary Figure 9d. Hence, the controller can learn from its own mistakes to progressively identify and distinguish different classes in general for realizing the meta-learning.

$$\boldsymbol{\lambda}_i = -\sum_{j=1}^{m} \left(\mathbf{Y}_{i,j} \log\left(\mathbf{P}_{i,j}\right) + \left(1 - \mathbf{Y}_{i,j}\right) \log\left(1 - \mathbf{P}_{i,j}\right) \right) \tag{3}$$

$$\text{loss} = \frac{1}{m} \sum_{i=1}^{b} \boldsymbol{\lambda}_i \tag{4}$$

$$\mathbf{Y} \in \mathbb{R}^{b \times m}, \quad \mathbf{P} \in \mathbb{R}^{b \times m}, \quad \boldsymbol{\lambda} \in \mathbb{R}^{b}$$

d. Episodic-training by repeating the above steps The three aforementioned steps form one training episode. Several hundreds or even thousands of such training episodes should be conducted so the model can perform well and provide meaningful predictions. Each episode is administered on a different (random) subset of the training classes. This prevents the model from overfitting. In the process, the parameters $\boldsymbol{\theta}$ of the embedding function $f_{\boldsymbol{\theta}}$ are updated such that objective function in Supplementary Equation 4 is minimized. This procedure is called maturing the controller.

A mature controller would be an optimally fitted embedding function, right at the verge of under- and overfitting. To avoid any overfitting, an early stopping[6] strategy can be

[2] For example, a particular version of stochastic gradient descent like "Adam"[4] works well.

applied. It relies on a subset of the training dataset kept aside as a validation set. As we are operating in the realm of few-shot learning, "a subset of the training set" implies non-overlapping classes. The validation set should be chosen large enough to properly represent the data but not too large as those samples are excluded from training.

During the training procedure, the model's performance is frequently evaluated on the validation set, without computing the loss and updating the controller's parameters. The performance can be measured with an accuracy metric computed per few-shot problem and states the fraction of correctly classified queries in a batch of size b:

$$\text{accuracy} = \frac{1}{b}\sum_{i=1}^{b}\left[1 \text{ if } \underset{j\in\{1,\ldots,m\}}{\operatorname{argmax}} \mathbf{P}_{i,j} = l_i \text{ else } 0\right]$$

A moderate number of queries b should be presented per problem and a rather large number of problems drawn from the validation set in order to average out fluctuations in the problem difficulty during evaluation. The state of the model yielding the best performance represents the mature controller.

2. Inference Phase

The outcome of the learning phase is the mature controller that is ready to learn and classify images from never-seen-before classes. During the inference phase, there is no update of the parameters of the mature controller (i.e., they are frozen), but the key-value memory will be updated by the controller upon encountering a new few-shot problem. The inference phase has two main steps similar to the learning phase: the support set loading step, and the query evaluation step. The first step generates support vectors from the forward pass through the mature controller, and writes them into the key memory followed by their labels into the value memory. This essentially leads to learning prototype vectors for the classes that are never exposed in the learning phase. By the end of this loading step, the key-value memory is *programmed* for the few-shot classification problem at hand. Then the query evaluation step similarly generates query vectors at the output of the controller that will be compared to the stored support vectors generating prediction labels. In a nutshell, if the backpropagation step in Supplementary Figure 9(d) is skipped and the support set and query batches are sampled from the test split instead of the train split, the sequence in Supplementary Figure 9 becomes similar to the inference phase (see also Supplementary Table 3).

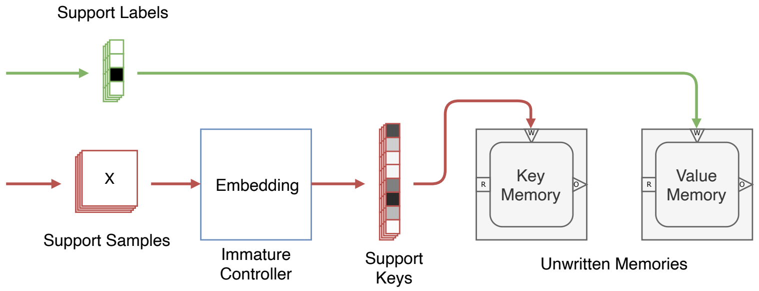

(a) Support set loading step.

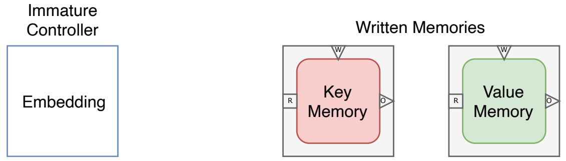

(b) State after the support set loading step.

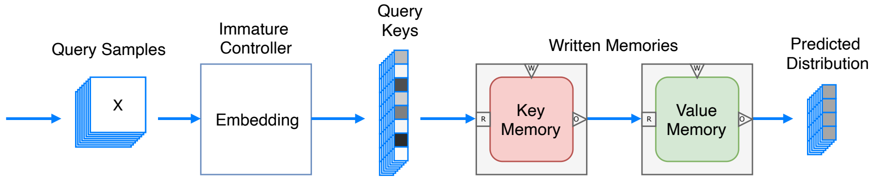

(c) Query evaluation step.

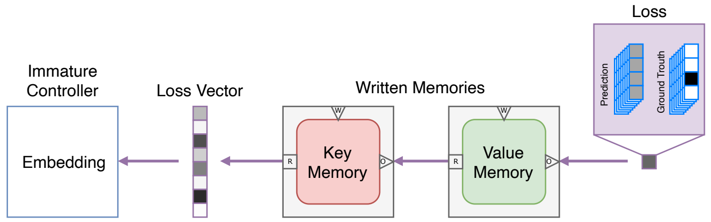

(d) Backpropagation step.

Supplementary Figure 9: Illustration of the learning phase with its steps.

Supplementary Note 3: Proof of the optimality of the sharpening function

We have the following functions in our training pipeline:

1. Embedding function used in the controller Eq 5, Eq 6.
2. Cosine similarity function measuring similarity between the query embedding $\mathbf{q}$ and key-memory embeddings $\mathbf{K}_{i,j}$ Eq 7.
3. Sharpening function applied on the similarity values Eq 8.
4. Prediction probabilities obtained by normalizing marginal sum of sharpened similarities Eq 9.
5. Loss function calculated as cross entropy between prediction probabilities and one hot encoded true label Eq 10.

$$\mathbf{q} = f(\mathbf{x}_q; \boldsymbol{\theta}) \tag{5}$$

$$\mathbf{K}_{i,j} = f(\mathbf{x}_{i,j}; \boldsymbol{\theta}) \tag{6}$$

$$\alpha_{i,j} = \frac{\mathbf{q} \cdot \mathbf{K}_{i,j}}{|\mathbf{q}|\,|\mathbf{K}_{i,j}|} \tag{7}$$

$$\varepsilon_{i,j} = \epsilon(\alpha_{i,j}) \tag{8}$$

$$P_j = \frac{\sum_{i=1}^{n} \varepsilon_{i,j}}{\sum_{j=1}^{m} \sum_{i=1}^{n} \varepsilon_{i,j}} \tag{9}$$

$$\lambda = -\sum_{j=1}^{m} \left(Y_j \log\left(P_j\right) + (1 - Y_j) \log\left(1 - P_j\right)\right) \tag{10}$$

$$i \in 1, 2, ..., n, \quad j \in 1, 2, ..., m, \quad \mathbf{q} \in \mathbb{R}^d, \quad \mathbf{K}_{i,j} \in \mathbb{R}^d,$$
$$\alpha_{i,j} \in [-1, 1], \quad P_j \in [0, 1], \quad Y_j \in \{0, 1\}, \quad \lambda \in \mathbb{R}$$

We aim to find optimum conditions for the sharpening function given in Eq 8. First, we seek the bounds for sharpened similarities $\varepsilon_{i,j}$.

Because prediction probabilities P_j must be non-negative $\rightarrow$ summed sharpened similarity $\sum_{i=1}^{n} \varepsilon_{i,j}$ for all m ways $\forall j$ should carry the same sign or it must be zero to satisfy Eq 9.
This condition can be met in two ways:

1. When $\varepsilon_{i,j} \geq 0 \qquad \forall i, j$
2. When $\varepsilon_{i,j} \leq 0 \qquad \forall i, j$

In this proof we focus on the first case, it can be similarly proved for the second case as well. This lets us set the bound for sharpened similarity as: $\varepsilon_{i,j} \in [0, \infty)$.

To further tighten this bound and infer other characteristics of the optimum sharpening function, we calculate partial derivative of loss λ given in Eq 10 w.r.t to P_j to find the optimum P_j that minimizes the loss.

$$\frac{\partial \lambda}{\partial P_j} = -\frac{Y_j}{P_j} + \frac{1 - Y_j}{1 - P_j} \tag{11}$$

The loss λ is minimized when $\frac{\partial \lambda}{\partial P_j} = 0$. By solving this we find optimum P_j as

$$P_j^* = Y_j \tag{12}$$

In other words,

$$P_j^* = \begin{cases} 1, & \text{if } j = j* \text{ is the true class index} \\ 0, & \text{otherwise} \end{cases} \tag{13}$$

In turn from Eq 9 we obtain the following constraints on ε that satisfies the optimum P_j^*:

$$\varepsilon_{max} = \max_{1 \le i \le n} \varepsilon_{i,j*} \tag{14}$$

$$\varepsilon_{min} = 0 \quad \text{when } j \neq j* \tag{15}$$

The domain of sharpening function ϵ is $\alpha \in [-1, +1]$ and our objective is to further tighten the bounds of the sharpening function. From Eq 14, 15, for the true class index $j*$, where cosine similarity between key memory embeddings and query are strong $\alpha_{i,j*} \to 1$ (see Eq 7), we get a new tight upper bound for the sharpened similarities as: $\varepsilon_{i,j} \in [0, \varepsilon_{max}]$ also a fixed point in the optimum sharpening function as: $\varepsilon\big|_{\alpha=1} = \varepsilon_{max}$.

For dissimilar classes $j = j' \neq j*$ where $\varepsilon_{min} = 0$, we would like to assign a cosine similarity value that can be learned within the range $-1 \le \alpha_{i,j'} \le 1$. We can immediately dismiss $\alpha_{i,j'} = 1$ because it is attained for the true class and we want to distinguish other classes from the true class. Then we consider $\alpha_{i,j'} = -1$. This anti-correlation state can be achieved only by a set of embeddings that all have the same unit vector direction. For 2-way problem this works, however, when the problem scales beyond 3-way problem, this is not feasible because two or more classes are forced to learn embeddings with the same unit vector which makes it difficult to distinguish those classes.

Next we consider $\alpha_{j_1,j_2} = 0$ (when $j_1 \neq j_2$). This yields zero cosine similarity (i.e. orthogonal) high-dimensional (HD) vectors for dissimilar classes allowing. As the dimensionality increases, we observe that the number of quasi-orthogonal vectors that can co-exist in a space increases exponentially. In HD computing theory, we observe that the maximum number of unique unit vectors with a fixed cosine similarity are obtained when pair-wise cosine similarity is zero i.e. $\alpha_{j_1,j_2} = 0$ (when $j_1 \neq j_2$), allowing us to learn a controller for the problem of highest possible number of classes for a given embedding dimensionality. With this, we fix another point in our optimum sharpening function as: $\varepsilon\big|_{\alpha=0} = \varepsilon_{min} = 0$ when $j \neq j*$.

To enable convergence towards this global minimum solution, we impose a monotonic increase on the positive axis of α i.e. $\alpha > 0$ and monotonic decrease when $\alpha < 0$ of the sharpening function. In order to ensure differentiability, the derivative at $\alpha = 0$ is set to be zero. This leads to the following further constraints:

$$\epsilon(\alpha_1) \le \epsilon(\alpha_2), \quad \text{when} \quad \alpha_1 \le \alpha_2, \quad \alpha_1 > 0, \quad \alpha_2 > 0 \tag{16}$$

$$\epsilon(\alpha_1) \ge \epsilon(\alpha_2), \quad \text{when} \quad \alpha_1 \le \alpha_2, \quad \alpha_1 < 0, \quad \alpha_2 < 0 \tag{17}$$

$$\frac{d\varepsilon}{d\alpha}\bigg|_{\alpha=0} = 0 \tag{18}$$

Although the above criteria are sufficient for the single shot learning case, we could add an additional constraint for the case of multi-shot learning. For example for a given dimensionality, the chance of the true class having a one shot with a similarity 0.6 maybe higher

than the true class having two shots both with 0.31 similarity. Furthermore the amount of noise that HD representations can tolerate depends on the chosen dimensionality: the larger the number of dimensions, the higher the robustness of the representations to the noise. Based on these observations, we can set a "private neighbourhood" similarity threshold i.e., those key memory embeddings that result in a similarity within the private neighbourhood are allowed to boost the prediction probability more than the ones outside this neighbourhood. In mathematical terms, this corresponds to a neighborhood of the inflection point(s) given by $\frac{d^2\varepsilon}{d\alpha^2} = 0$ in the sharpening function.

Based on the above constraints and criteria we can visualize the family of optimum sharpening functions as given in Figure 10.

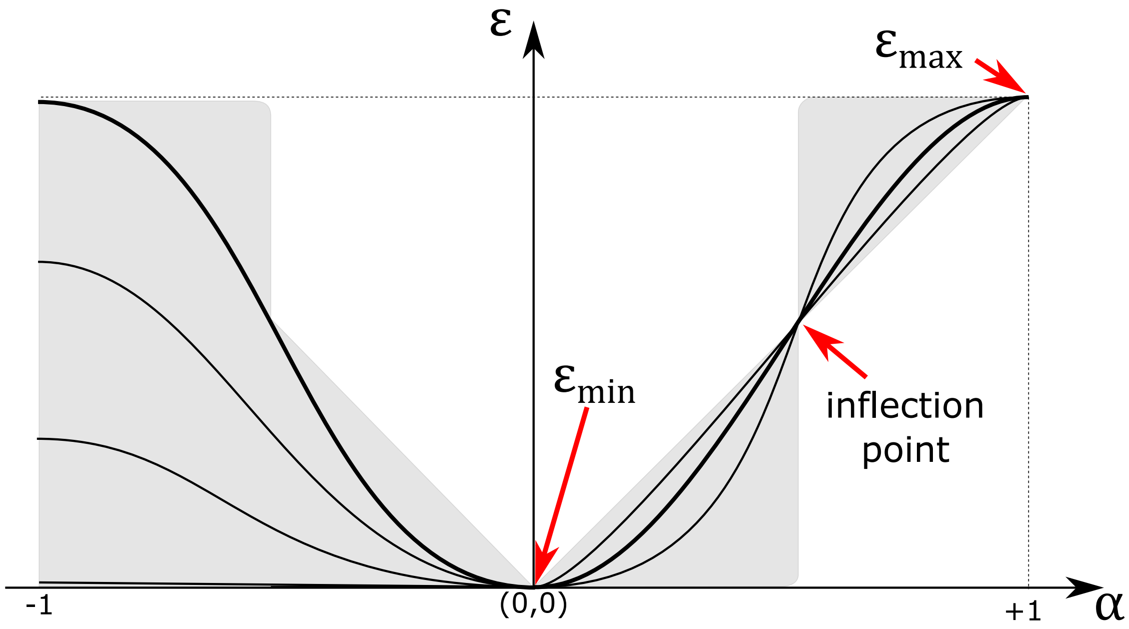

Supplementary Figure 10: Visualization of family of optimum sharpening functions. The softabs function is highlighted in bold

The softabs sharpening function that we propose is:

$$\epsilon(\alpha) = \frac{1}{1 + e^{-(\beta(\alpha-0.5))}} + \frac{1}{1 + e^{-(\beta(-\alpha-0.5))}} \tag{19}$$

This function readily meets the optimal conditions given in Eq 14, 16, 17, 18, in addition to setting the inflection points $\frac{d^2\varepsilon}{d\alpha^2} = 0$ at -0.5 and 0.5, whereas the softmax sharpening function does not have any inflection points and fails to meet the conditions in Eq 15, 17, 18. Although the softabs does not exactly meet the condition in Eq 15, it approximates this condition very closely. For example, for $\beta = 10$ we get epsilon(0)=0.0134 which leads to a $27\times$ better approximation than the softmax.

Supplementary Note 4: Comparison of classification accuracy between sum-argmax vs argmax as the ranking criteria

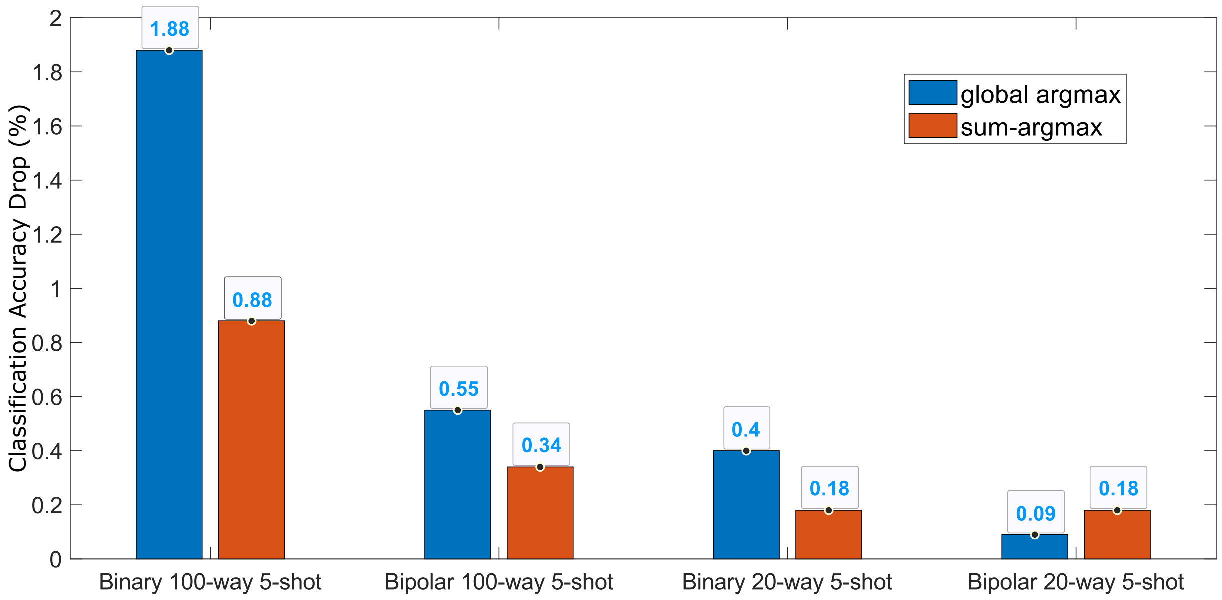

Supplementary Figure 11: Comparing classification accuracy drop from the ideal crossbar to the PCM experiment based on the sum-argmax vs global-argmax across different problems each from 1000 testing episodes of a single run. In all problems except bipolar 20-way 5-shot problem, sum-argmax results in lower accuracy drop, indicating its robustness as the ranking criterion.

We investigate two different selection criteria to choose the final predicted class from the produced attention vector during inference. We first consider the typical argmax of the components across the whole attention vector ($\mathbf{w}$). We call this the *global argmax* and it returns the label of a support vector, among all the mn support vectors, whose probability is the highest. As the second criterion, we propose sum-argmax where the same class components of the attention vector are first summed together before applying the argmax function on m summed values (i.e., the number of classes). For an m-way n-shot problem, the global argmax has $\mathcal{O}(nm)$ comparison operations, while the sum argmax has $\mathcal{O}(m)$ comparisons together with $\mathcal{O}(mlog(n))$ addition operations; these operations are much fewer than the number of operations involved in the key memory for the similarity search with the high-dimensional vectors. Hence, the computational complexity of the selection criterion is not dominant, and does not affect the overall computational complexity of the key-memory.

However, as shown in Supplementary Figure 11, the sum-argmax exhibits a clear advantage over the global argmax, in mitigating the accuracy drop in the presence of noise when the key memory is implemented on the PCM devices, as opposed to the ideal software model without any variations. This lower accuracy drop is observed for both bipolar and binary representations, especially in the large problem sizes. For instance, the 100-way 5-shot problem with the binary representation reaches up to 1% better accuracy mitigation by using the sum-argmax instead of the global argmax function. This is mainly due to the fact that in the sum-argmax function the variations among various classes programmed on

the PCM array can be better averaged out by adding the intra-class probabilities. As a further observation, the sum-argmax, which yields a more robust ranking criterion, cannot be implemented in the TCAM-based architectures because the TCAM can only compute argmax as the speed of a matchline discharge.

Supplementary Note 5: Effect of approximating sharpening function and similarity function

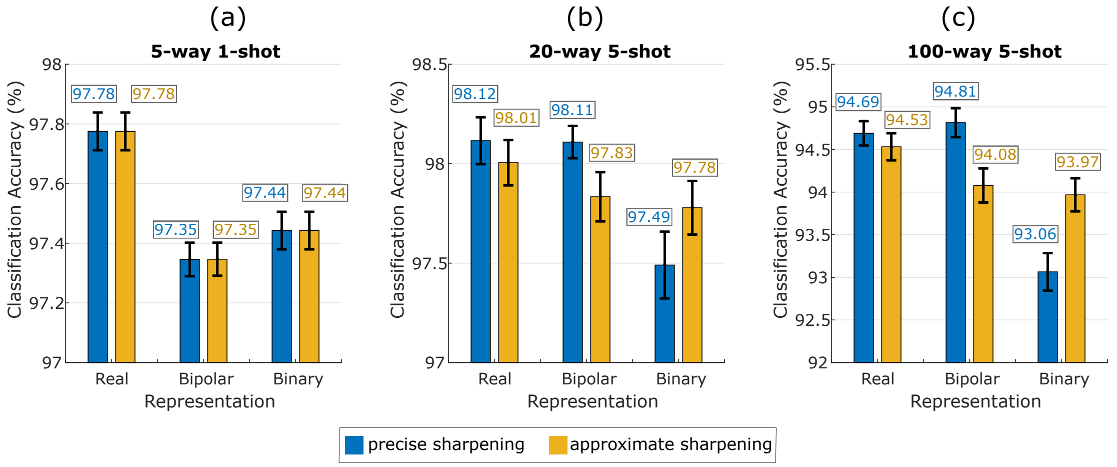

Supplementary Figure 12: Classification accuracy comparison between approximate sharpening function and precise sharpening function for three problems: 5-way 1-shot(a) 20-way 5-shot(b) 100-way 5-shot(c), from 10 independent runs each with 1000 episodes. For all the problems, the precise similarity function is used. The error bars represent one standard deviation of sample distribution on either directions.

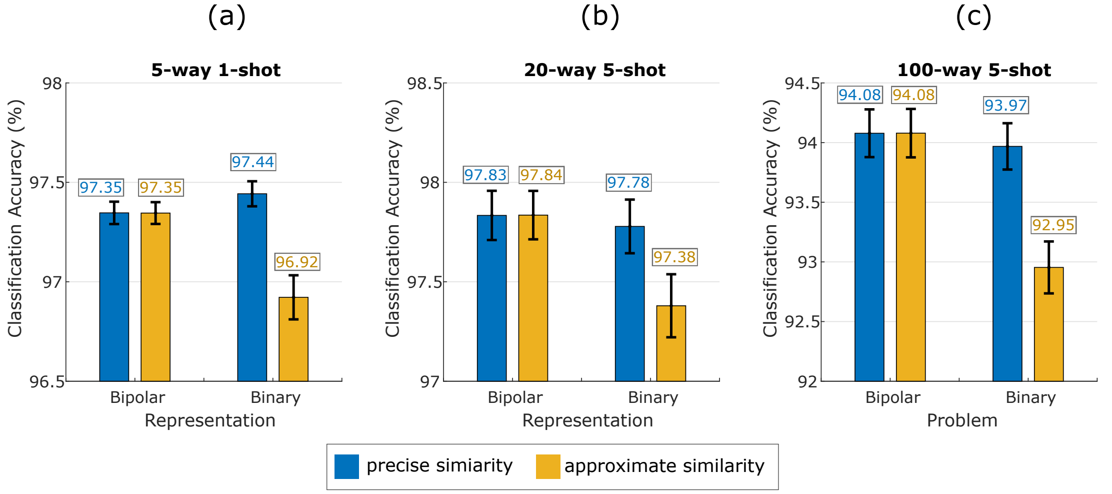

Supplementary Figure 13: Classification accuracy comparison between approximate similarity function and precise similarity function for three problems: 5-way 1-shot(a) 20-way 5-shots(b) 100-way 5-shots(c) from 10 independent runs each with 1000 episodes. For all the problems, the approximate sharpening function is used. The error bars represent one standard deviation of sample distribution on either directions.

Supplementary Figure 12 shows the impact of using the softabs as the precise sharpening function versus the regular absolute function as the approximate version, during inference for

three different representations. As shown, the softabs function consistently reaches a higher accuracy in both the real and bipolar representations across all the three problems. However, the softabs has a negative impact on the accuracy of binary representation. Therefore, using the regular absolute function, which in fact can be bypassed in the binary representation, not only simplifies the inference architecture but also slightly improves the accuracy.

Supplementary Figure 13 shows the impact of using the cosine similarity as the precise similarity metric versus the dot product as the approximate version. The approximate sharpening functions are used for each configuration. A significant accuracy drop is observed in the binary case when the cosine similarity is approximated by dot product. This drop is due to the fact that the controller produces binary vectors that do not have a fixed norm but a norm approximately close to $\sqrt{\frac{d}{2}}$, whereas this is not the case for the bipolar vectors with a constant norm of $\sqrt{d}$. When the similarity metric is not approximated, the binary is on average better than the bipolar, indicating that the similarity approximation is the cause of accuracy drop in the case of the binary representation. The results are collected from 10 independent runs from each configuration.

Supplementary Note 6: Spatial Variability on PCM Crossbar

The particular conductance levels determine the power consumption of the crossbars during read-outs and the variability of the programmed states. Usually, the lower the conductance level of the state, the larger the deviations. The conductance of the RESET state for such devices is usually low enough so that currents cannot be detected by the analog-to-digital converter during read-out. Since our binary representations are extremely robust against deviations, we used low and thus power-saving conductance levels for the SET state of the key memory crossbar. Supplementary Figure 14(a) illustrates typical SET variations encountered at programming time of the prototype chip used for the experiments.

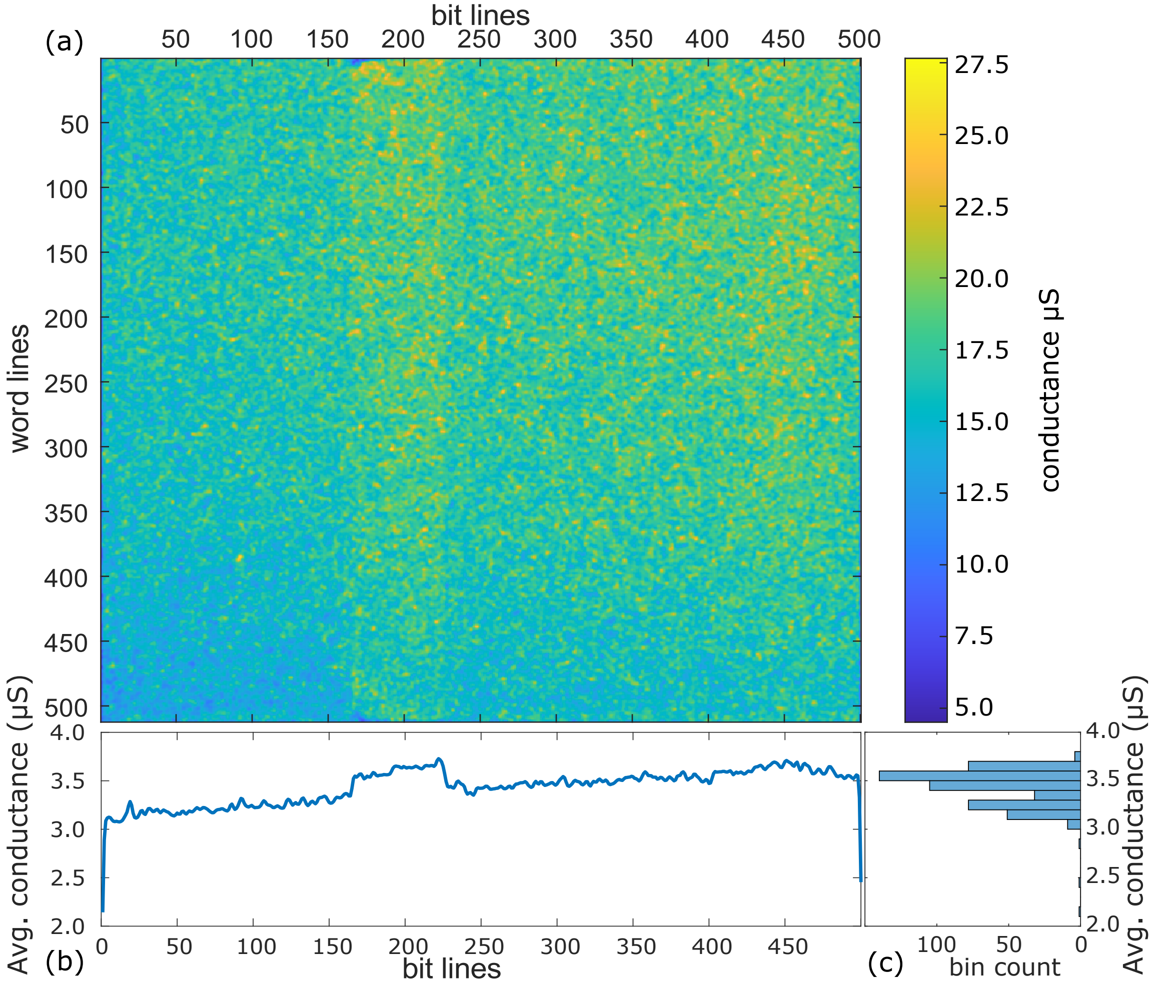

Supplementary Figure 14: (a) Spatial variability of SET state conductance of device across the PCM array. (b) Average SET state conductance per bitline. (c) Distribution of Average SET state conductance per bitline.

The relative standard deviation of the spatial variability of the set state conductance (σ_{rel}) at programming timescales is observed as 31.7% across the entire region of the chip utilized for 100-way 5-shot problem. When SET state conductance is averaged per bitline there is 5.38% relative standard deviation in the average SET state conductance across bit lines as shown in Supplementary Figure 14(b) and (c). This spatial variability is the cause for further classification accuracy loss of 0.11% in PCM experiments when compared with

PCM model simulations with the same parameters as with the prototype chip used for the experiments.

Supplementary Note 7: Accuracy comparison with the state-of-the-art few-shot learning models

Here, we compare classification accuracy of our MANN model against the state-of-the-art few-shot learning models based on meta-learning approach. The range of relevant models can be broadly divided into three different settings:

1. **Type I**: Purely software models, using the cosine similarity and the highest precision, aimed to run on conventional CPUs/GPUs. The key memory vector components are represented by 32-bit real numbers. As shown in Supplementary Table 4, among all the models, the Matching Networks[7], generally provides the highest accuracy. At maximum, it achieves 0.49% higher accuracy compared to our model for the 20-way 5-shot problem, but it used an unconventional split of training and evaluation in Omniglot dataset that led to including 4720 more training examples from 236 classes. To show the scalability of our approach, we also extended the repertoire of standard Omniglot problems up to 100-way 5-shot. For this largest problem ever-tried on the Omniglot, the accuracy of our model is still in the range of problems with a smaller number of ways. There is no report from other models on this large problem size.

2. **Type II**: Models simulated with an ideal low-precision key memory. In this setting, we consider a deterministic (i.e., without noise) behavior in the hardware such that the accuracy degradation due to the device non-ideality and noise are not included. In our model, the key memory uses the binary representation (1-bit per vector component), while the other works assume an optimal 4-bit representation[1], or a 3-bit representation[3]. Targeting the PCM hardware, we employ an approximation to the common cosine similarity computation as mentioned in the paper, whereas the other works consider other distance metrics such as combined $L_1 + L_\infty$ distance (for which only L_∞ can be implemented in TCAM and L_1 is computed outside the crossbar[1]), or a modified version targeting multibit CAM[3]. Despite working with the lowest precision of 1-bit, our model with the softabs sharpening function provides the highest accuracy across all the problems as shown in Supplementary Table 4.

3. **Type III**: Models simulated with a noisy and low-precision key memory. In this setting, the key memory aimed to withstand noise and stochastic variabilities of in-memory computing hardware. We simulate our model with 30% conductance variation that we observed in the experimental PCM platform. This we compare against the multibit CAM model[3] with its threshold voltage variation at its lowest state S1 equal to 30%, which amounts to less than 30% variation for the other higher state S2,...,S8. Despite the higher relative variation, our model achieves higher accuracy across all the problems showing its robustness even with binary representations.

In another column in Supplementary Table 4, we also compare the classification accuracy of our model with the softmax sharpening function. The results show that the accuracy gap between the softmax and the sofabs can be as high as 19.26% in the software model. Although the softmax works with the same vector dimensionality as the softabs, the results demonstrate that simply expanding the vector dimensionality to HD space is not sufficient, and therefore there is a need for the proper sharpening function to direct the vector representations during training. Further, the resulting vector representations by the softmax is less robust than those obtained by the softabs: e.g., in the 100-way 5-shot problem, there is

4.09% accuracy drop by going from the software (Type I) to the noisy hardware (Type III) with the softmax, while the softabs causes only 2.46% accuracy drop.

Supplementary Table 4: Comparison of classification accuracy with the state-of-the-art models at three different settings: software, simulated in-memory hardware without noise, and simulated in-memory hardware with noise. Th key memory in our model uses 32-bit floating-point vectors in the software setting, and binary vectors in both hardware settings.

reference	[5]	[1]	[3]	[7]	This (softmax)	**This (softabs)**
**Type I:** 32-bit real numbers and cosine similarity in software						
5-way 1-shot	96.59*	96.59*	96*	**98.1***	94.14	97.78
20-way 1-shot	-	**95***	89*	93.8*	84.67	94.61
20-way 5-shot	-	98.5*	97*	**98.5***	90.19	98.01
100-way 5-shot	-	-	-	-	75.27	**94.53**
**Type II:** 1–4-bit in-memory hardware simulations without noise						
5-way 1-shot	-	24.99*	95*	-	93.06	**97.44**
20-way 1-shot	-	-	89*	-	82.32	**93.18**
20-way 5-shot	-	-	95*	-	88.96	**97.78**
100-way 5-shot	-	-	-	-	73.44	**93.97**
**Type III:** 1–3-bit noisy in-memory hardware simulations (with maximum of 30% σ_{rel})						
5-way 1-shot			92*		93.06	**96.40**
20-way 1-shot			81*		82.31	**93.19**
20-way 5-shot			93*		88.96	**97.60**
100-way 5-shot					71.18	**92.07**

*Accuracy obtained from an unconventional training and evaluation split of the Omniglot dataset

Supplementary Note 8: Proof of robustness of noisy cosine similarity with binary high-dimensional vectors

Let us take two binary high-dimensional vectors $\hat{\mathbf{a}}$ and $\hat{\mathbf{b}}$, which fulfill the property $\|\hat{\mathbf{x}}\| \approx \sqrt{\frac{d}{2}}$. We write $\hat{\mathbf{b}}$ to the computational memory and use $\hat{\mathbf{a}}$ as the readout voltage. Thus, the latter remains accurate whereas the former (written into the memory) has to be modeled as a vector of normal random variables $\hat{\mathbf{B}}$ with

$$\hat{\mathbf{B}}_i = X \text{ if } \hat{\mathbf{b}}_i = 1, \text{ else } 0$$
$$\mathrm{E}(X) = 1, \quad \mathrm{Var}(X) = \sigma_{\text{rel}}^2.$$

In the next step, we consider the (approximate) cosine similarity between the original vectors a fixed value α and rearrange its expression:

$$\begin{aligned}
\alpha = \frac{2}{d}\,\hat{\mathbf{a}} \cdot \hat{\mathbf{b}} &= \frac{2}{d}\sum_{i=1}^{d} \hat{\mathbf{a}}_i \cdot \hat{\mathbf{b}}_i \\
&= \frac{2}{d}\sum_{i=1}^{d} (1 \text{ if } \hat{\mathbf{a}}_i = \hat{\mathbf{b}}_i = 1) \\
&= \frac{2}{d}\sum_{i=1}^{n} 1 = \frac{2n}{d},
\end{aligned}$$

where n denotes the number of positions where both $\hat{\mathbf{a}}$ and $\hat{\mathbf{b}}$ equal 1. Analogously, we obtain

$$\begin{aligned}
\Lambda = \frac{2}{d}\,\hat{\mathbf{a}} \cdot \hat{\mathbf{B}} &= \frac{2}{d}\sum_{i=1}^{d} (X \text{ if } \hat{\mathbf{a}}_i = \hat{\mathbf{b}}_i = 1) \\
&= \frac{2}{d}\sum_{i=1}^{n} X,
\end{aligned}$$

where the random variable Λ denotes the result of the noisy cosine similarity operation.

Applying the rules of probability theory, we compute the expected value and standard deviation of Λ as

$$\begin{aligned}
\mathrm{E}(\Lambda) &= \mathrm{E}\left(\frac{2}{d}\sum_{i=1}^{n} X\right) & \mathrm{Var}(\Lambda) &= \mathrm{Var}\left(\frac{2}{d}\sum_{i=1}^{n} X\right) \\
&= \frac{2}{d}\sum_{i=1}^{n} \mathrm{E}(X) & &= \left(\frac{2}{d}\right)^2 \sum_{i=1}^{n} \mathrm{Var}(X) \\
&= \frac{2n}{d} = \alpha & &= \left(\frac{2}{d}\right)^2 n\sigma_{\text{rel}}^2 = \left(\frac{2}{d}\right)^2 \frac{\alpha d}{2}\sigma_{\text{rel}}^2 = \frac{2\alpha\sigma_{\text{rel}}^2}{d}.
\end{aligned}$$

This finally leads to

$$\sigma(\Lambda) = \sqrt{\frac{2\alpha}{d}}\sigma_{\text{rel}}.$$

This states that the standard deviation of the cosine similarity is inversely proportional to the square root of the dimensionality, and is thus able to diminish the influence of the large SET state variability. Furthermore, a deviation of the expected value $\mathrm{E}(X)$ from 1 has a direct influence on the expected value of Λ:

$$\mathrm{E}(\Lambda) = \varepsilon\alpha \quad \text{when} \quad \mathrm{E}(X) = \varepsilon.$$

Therefore, all crossbar devices' SET state should exhibit the same mean, which otherwise can only be compensated by the quality of the representations themselves.

SUPPLEMENTARY REFERENCES

[1] Ann Franchesca Laguna, Michael Niemier, and X. Sharon Hu. Design of Hardware-FriendlyMemory Enhanced Neural Networks. In *Design, Automation Test in Europe Conference Exhibition (DATE)*, pages 1583–1586, 2019.

[2] P. Kanerva. Hyperdimensional computing: An introduction to computing in distributed representation with high-dimensional random vectors. *Cognitive Computation*, 1(2):139–159, Jun 2009.

[3] A. Kazemi, M. M. Sharifi, A. F. Laguna, F. Müller, R. Rajaei, R. Olivo, T. Kämpfe, M. Niemier, and X. S. Hu. In-Memory Nearest Neighbor Search with FeFET Multi-Bit Content-Addressable Memories. *Preprint at* `http://arxiv.org/abs/2011.07095`, 2020.

[4] D. Kingma and J. Ba. Adam: A method for stochastic optimization. *International Conference on Learning Representations*, 2014.

[5] A. F. Laguna, X. Yin, D. Reis, M. Niemier, and X. S. Hu. Ferroelectric FET based in-memory computing for few-shot learning. In *Proceedings of the ACM Great Lakes Symposium on VLSI*, pages 373–378, 2019.

[6] L. Prechelt. Early stopping — but when? In *Neural Networks: Tricks of the Trade: Second Edition*, pages 53–67. 2012.

[7] O. Vinyals, C. Blundell, T. Lillicrap, K. Kavukcuoglu, and D. Wierstra. Matching Networks for One Shot Learning. *Preprint at* `http://arxiv.org/abs/1606.04080`, 2016.
